# Genetic mapping of putative *Chrna7 *and *Luzp2 *neuronal transcriptional enhancers due to impact of a transgene-insertion and 6.8 Mb deletion in a mouse model of Prader-Willi and Angelman syndromes

**DOI:** 10.1186/1471-2164-6-157

**Published:** 2005-11-09

**Authors:** Mihaela Stefan, Kathryn C Claiborn, Edyta Stasiek, Jing-Hua Chai, Tohru Ohta, Richard Longnecker, John M Greally, Robert D Nicholls

**Affiliations:** 1 Center for Neurobiology and Behavior, Department of Psychiatry, University of Pennsylvania, Philadelphia, PA 19104, USA; 2 Department of Genetics, University of Pennsylvania, Philadelphia, PA 19104, USA; 3 Division of Hematology, Department of Medicine, Albert Einstein College of Medicine, The Bronx, USA; 4 Department of Microbiology and Immunology, Feinberg School of Medicine, Northwestern University, Ward 6-231, 303 East Chicago Ave, Chicago, IL 60611, USA; 5 Birth Defects Laboratories, Children's Hospital of Pittsburgh, Room 2109 Rangos Research Center, 3460 Fifth Avenue, Pittsburgh, PA 15213, USA; 6 Health Science University of Hokkaido, Hokkaido, Japan; 7 Department of Pediatrics, Children's Hospital of Pittsburgh, 3460 Fifth Avenue, Pittsburgh, PA 15213

## Abstract

**Background:**

Prader-Willi and Angelman syndrome (PWS and AS) patients typically have an ~5 Mb deletion of human chromosome 15q11-q13, of opposite parental origin. A mouse model of PWS and AS has a transgenic insertion-deletion (TgPWS/TgAS) of chromosome 7B/C subsequent to paternal or maternal inheritance, respectively. In this study, we define the deletion endpoints and examine the impact on expression of flanking genes.

**Results:**

Using molecular and cytological methods we demonstrate that 13 imprinted and 11 non-imprinted genes are included in the TgPWS/TgAS deletion. Normal expression levels were found in TgPWS brain for genes extending 9.1- or 5.6-Mb centromeric or telomeric of the deletion, respectively. Our molecular cytological studies map the proximal deletion breakpoint between the *Luzp2 *and *Siglec-H *loci, and we show that overall mRNA levels of *Luzp2 *in TgPWS and TgAS brain are significantly reduced by 17%. Intriguingly, 5' *Chrna7 *shows 1.7-fold decreased levels in TgPWS and TgAS brain whereas there is a ≥15-fold increase in expression in neonatal liver and spleen of these mouse models. By isolating a *Chrna7*-Tg fusion transcript from TgAS mice, we mapped the telomeric deletion breakpoint in *Chrna7 *intron 4.

**Conclusion:**

Based on the extent of the deletion, TgPWS/TgAS mice are models for PWS/AS class I deletions. Other than for the first gene promoters immediately outside the deletion, since genes extending 5.6–9.1 Mb away from each end of the deletion show normal expression levels in TgPWS brain, this indicates that the transgene array does not induce silencing and there are no additional linked rearrangements. Using gene expression, non-coding conserved sequence (NCCS) and synteny data, we have genetically mapped a putative *Luzp2 *neuronal enhancer responsible for ~33% of allelic transcriptional activity. The *Chrna7 *results are explained by hypothesizing loss of an essential neuronal transcriptional enhancer required for ~80% of allelic *Chrna7 *promoter activity, while the *Chrna7 *promoter is upregulated in B lymphocytes by the transgene immunoglobulin enhancer. The mapping of a putative *Chrna7 *neuronal enhancer inside the deletion has significant implications for understanding the transcriptional regulation of this schizophrenia-susceptibility candidate gene.

## Background

Prader-Willi and Angelman syndrome (PWS and AS) are complex neurobehavioral disorders associated with loss of function of a cluster of differentially expressed imprinted genes in chromosome 15q11-q13 [1]. PWS is characterized by a neonatal stage of failure to thrive, hypotonia and respiratory distress followed by hyperphagia in early childhood with development of severe obesity, as well as short stature, hypogonadism, small hands and feet, mild to moderate mental retardation, and obsessive-compulsive behavior [2,3]. In contrast, AS patients have a more pronounced neurological disease including developmental delay, severe mental retardation with lack of speech, hyperactivity, seizures, aggressive behavior and excessive inappropriate laughter [2]. Most PWS and AS cases (~70%) are due to ~5 Mb *de novo *deletions spanning a 2 Mb imprinted domain and several adjacent non-imprinted genes [1]. There are two classes of deletions in PWS/AS patients, one from breakpoint 1 (BP1) to BP3 and the other from BP2 to BP3 [4]. Additionally, paternal or maternal uniparental disomy (pat or matUPD) explain 25% of PWS and 5% of AS cases, respectively, while 2–5% of PWS and AS cases result from imprinting defects (ID). In each mechanism, PWS arises from loss of ten paternally expressed loci, while AS arises from loss of function of the maternally expressed *UBE3A *gene [1].

**Figure 1:**
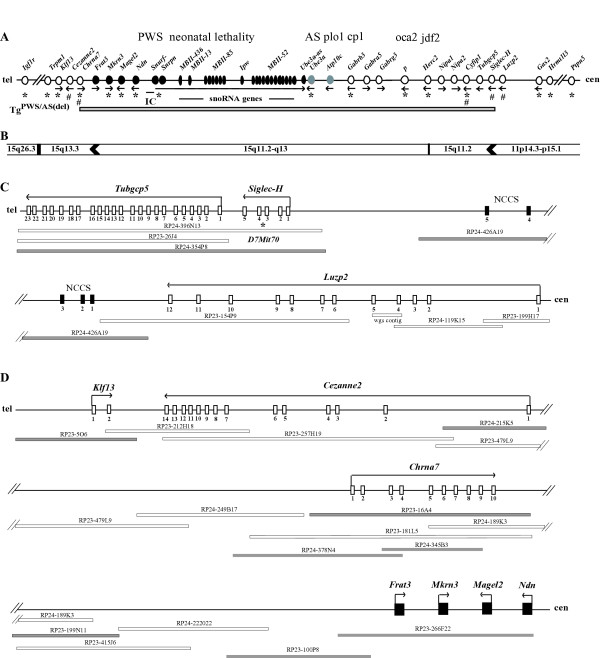
**Genetic and physical maps of mouse chromosome 7B/C**. **(A) The mouse PWS/AS-homologous region and flanking genes**. Symbols are: circles, protein-coding genes; ovals, RNA-coding genes; black, paternally-expressed; grey, maternally-expressed; white, biparentally-expressed; line arrows, transcriptional orientation of genes; IC; imprinting center; plo1, *p*-locus-associated obesity; cp1, cleft palate 1; oca2, oculocutaneous albinism type II (*p*, pink-eyed dilution); jdf2, juvenile development and fertility 2; cen, centromere; tel, telomere; *, genes represented on the Gene chip MG_U74Av2 (see Table 1); #, genes analyzed by QRT-PCR; horizontal grey bar, extent of the TgPWS/TgAS ~5 Mb deletion. The first 3 genes and one more distant gene extending out from each of the centromeric and telomeric deletion breakpoints are also shown. **(B) Human synteny for genes from the mouse chromosome 7B/C domain**. The human chromosome locations for each orthologous mouse gene shown in (A) are given. Arrowheads represent rodent-specific gene duplications for which no ortholog is found in human. **(C) BAC contig across the TgPWS/TgAS centromeric deletion breakpoint region**. Symbols are: white boxes, exons; line arrows, transcriptional orientation of genes; white bars, BACs, and one "wgs" (whole genome shotgun) contig; grey bars, BACs used as FISH probes; black boxes, Non-Coding Conserved Sequences (NCCSs) in RP24-246A19; *, *D7Mit70*. The interval from *Luzp2 *through *Siglec-H *and *Tubgcp5 *is shown. **(D) BAC contig across the TgPWS/TgAS telomeric deletion breakpoint region**. Symbols are as for Fig 1C, except: black boxes, four intronless imprinted genes. The interval from the telomeric end of the 2 Mb imprinted gene domain through *Chrna7 *to *Cezanne2 *and *Klf13 *is shown.

Mouse models of PWS with either matUPD [5], an ID [6] or a paternally-inherited chromosome deletion [7] share a similar phenotype with failure to thrive, hypotonia and early postnatal lethality, modeling the first stage of the human syndrome [9,10]. Similarly, mouse models of AS have a patUPD [10], maternally-inherited chromosome deletion [7], or a maternal mutation of *Ube3a *[11,12]. In the transgenic (Tg) deletion mouse model, an Epstein Barr Virus *LMP2A *transgene integrated with ~80 copies into mouse chromosome 7B/C and created an ~5 Mb deletion of the mouse region homologous to the human PWS/AS genes (see Fig. 1A,B) [7]. As in human, the phenotype of the deletion mouse model depends on the parental origin: paternal or maternal inheritance of the Tg-deletion, respectively, results in the TgPWS mouse model characterized by severe neonatal hypoglycemia and early lethality [9] or in TgAS mice with a mild neurobehavioral phenotype and late onset obesity [7].

Previous imprinted gene expression studies and fluorescence *in situ *hybridization (FISH) showed that the Tg insertion-deletion comprised all of the orthologs of PWS and AS imprinted genes and of several flanking non-imprinted genes [Fig. 1A; [7,13,14]]. In addition, brain microarray and quantitative gene expression analyses confirmed the loss of expression of several imprinted genes (*Snurf-Snrpn*, *Ndn*, *Magel2*, *Mkrn3*) and 50% reduced expression of two non-imprinted loci (*Herc2*, *Cyfip1*) in TgPWS mouse brain, and demonstrated that an unidentified non-imprinted locus within the deletion acted *in trans *to regulate a chromosome 18B3 gene expression domain [15]. To define the exact characteristics of the transgene insertion-deletion TgPWS/TgAS mouse model, we have now determined the extent of the deletion and the effect of the Tg-insertion and deletion on expression of flanking genes. We delineated the deletion breakpoint positions between *Siglec-H *and *Luzp2 *at the centromeric end and within intron 4 of *Chrna7 *at the telomeric end. Most importantly, we describe tissue-specific positional effects of the Tg insertion and/or deletion on *Luzp2 *and *Chrna7 *expression which are likely due to the presence or absence of specific enhancer elements.

## Results

### FISH refines the TgPWS/TgAS deletion extent and identifies a centromeric breakpoint

**Figure 2:**
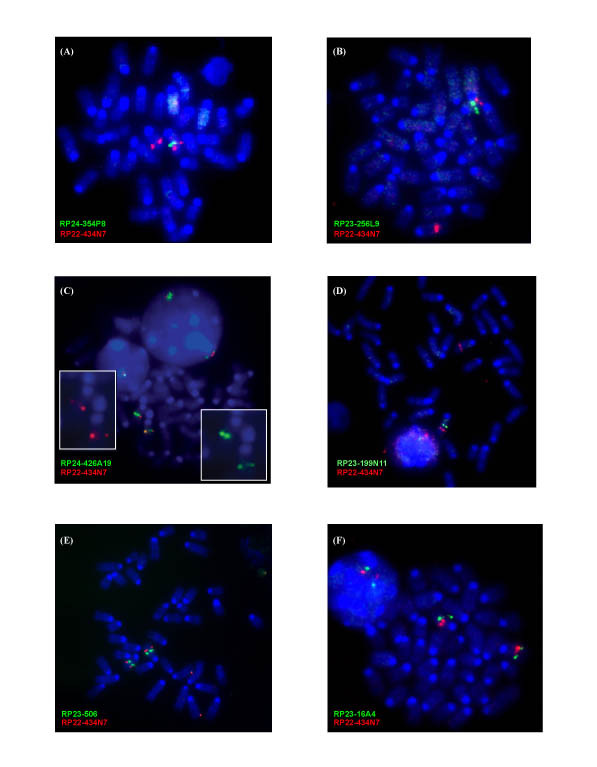
**Fluorescence *in situ *hybridization (FISH) maps the TgAS centromeric and telomeric deletion extent**. In each case, BAC RP22-434N7 from the chromosome 7 Tyrosinase (*Tyr*) locus (74.6 Mb; 44.0 cM) was hybridized as a control and is shown as a red signal. All chromosome 7B/C BACs used as probes are shown as green signals while chromosomes are stained with DAPI (blue). **(A) BAC RP24-354P8 **spanning the region including *Siglec-H *and *Tubgcp5 *shows a single-chromosome 7 signal indicating the deletion of this locus in TgAS splenocytes. **(B) RP23-256L9 **spans the *p *locus (exons 10–24) and is also deleted in TgAS mice. **(C) RP24-426A19 **spans the region 3' of *Luzp2 *and detects a weak signal on one chromosome 7 homologue, suggesting partial deletion and detection of the centromeric breakpoint in the mutant mice. The two insets show the images for individual probes. **(D) RP23-199N11 **covers part of the region between *Frat3 *and *Chrna7 *and is deleted in TgAS mice. **(E) RP23-506 **for the *Klf13 *locus is intact in the deletion mice. **(F) RP23-16A4 **spans all 10 exons of *Chrna7 *and shows an apparently intact signal in TgAS mice.

We used FISH with BAC probes flanking the mouse PWS/AS-homologous domain in chromosome 7B/C (Fig. 1C,D) to define the centromeric and telomeric extent of the TgPWS/TgAS deletion. To identify both chromosome 7 homologues in our FISH experiments using splenocytes from TgAS mice or fibroblasts from TgPWS mice, we co-hybridized BAC RP22-434N7 from the *Tyr *gene locus at position 74.6 Mb (chromosome 7E1), which is intact in TgPWS and TgAS mice (Fig. 2, and data not shown) [7].

At the centromeric end of the deletion, BAC RP24-354P8 spanning the *Siglec-H*-*D7Mit70*-*Tubgcp5 *region (43.3 Mb, Fig. 1C) is deleted from one chromosome 7 in TgAS mice (Fig. 2A). Additionally, BACs RP23-256L9 (*p *gene exons 10–24; 44 Mb) and RP23-195C6 (*Herc2*-*Nipa1*; 43.6 Mb) are within the TgPWS/TgAS deletion (Fig. 1A, Fig. 2B, and data not shown) as expected based on their map position. In contrast, BAC RP24-426A19 from ~50 kb 3' of *Luzp2 *(42.9 Mb; Fig. 1C) shows a weak positive signal on one chromosome 7 homologue in TgAS mice (Fig. 2C), suggesting a partial deletion and detection of the centromeric breakpoint in TgAS mice.

At the telomeric end of the deletion, BAC RP23-266F22 (49.4 Mb; Fig. 1D) spanning four mouse PWS-region imprinted genes, including *Frat3*, *Mkrn3*, *Magel2 *and *Ndn*, was previously found to be deleted in TgPWS mice [13]. Extending telomeric from *Frat3 *towards *Chrna7 *(Fig. 1D), BACs RP23-100P8, RP23-199N11 and RP23-140J4 were all deleted in TgPWS mice (Fig. 2D, and data not shown). In contrast, BACs RP23-5O6 from the *Klf13 *region (50.9 Mb; Fig. 1D, Fig. 2E) and RP24-215K5 from 5' *Cezanne2 *(Fig. 1D; data not shown) were intact in TgPWS/TgAS mice. Two other BACs mapping more telomeric, RP23-76F18 (*Rlbp1 *locus, 66.4 Mb) and RP23-441D12 (*Il-16 *locus, 70.8 Mb), were also intact in TgPWS mice (data not shown). Intriguingly, BAC RP23-16A4 spanning *Chrna7 *(50.1 Mb; Fig. 1D) appears based on hybridization signal intensity to be fully intact in TgPWS mice (Fig. 2F). Nevertheless, molecular data described below indicate that about half of 16A4 is deleted in TgPWS/TgAS mice; it is likely that these ostensibly contradictory data are explained by the relative concentration of unique and repetitive DNA sequences at the telomeric and centromeric ends of BAC 16A4 (5' and 3' ends of *Chrna7*, respectively) as well as the difficulty in quantifying FISH signals.

### Microarray and QRT-PCR analyses further define the TgPWS/TgAS deletion extent

Previous analysis of brain global gene expression in 5 TgPWS and 5 wildtype (WT) mice at P1 using a MG-U74Av2 gene chip array (Affymetrix) which assayed 12,000 genes and ESTs, demonstrated that all 4 paternally-expressed mouse PWS-region imprinted genes present on the array (*Snurf-Snrpn*, *Ndn*, *Magel2 *and *Mkrn3*) have dramatically reduced expression [15]. Additionally, mRNA levels for non-imprinted genes *Herc2 *and *Cyfip1 *were 50% in TgPWS compared with WT [15]. To assess the extent of the TgPWS/TgAS deletion and the potential effect of the transgene-insertion deletion on expression of flanking genes, we used data mining from several genome databases and analysis of several megabases of genome sequence to identify all genes in these regions, determined which of these were on the MG-U74Av2 gene chip, and then reanalyzed the brain microarray data from reference [15]. Although a 3–4 Mb "gene desert" lies just proximal of *Tubgcp5*, we identified 9 genes on the chip in the 9.1 Mb region centromeric of *Tubgcp5*, a locus which FISH data above had shown was deleted, of which four genes were not detectable in either TgPWS or WT brain at P1 (Table 1). Five genes (*Gas2*, *Hrmt1l3*, *E2F8*, *Ptpn5*, and *Tsg101*) were detectable and showed no change in TgPWS compared to WT mouse brain (Table 1), indicating that the TgPWS/TgAS deletion does not affect any of these genes (Fig. 1A). Telomeric of *Frat3*, an imprinted gene deleted in TgPWS mice [13], nine (*Klf13*, *BB128963*, *Mcee*, *Apba2*, *Tjp1*, *Snrp2a*, *H47*, *Mef2a*, and *Igf1r*) of the fourteen genes extending out a further 5.6 Mb and on the microarray were detectable and all had comparable levels in TgPWS and WT mice (Table 1). Therefore, the Tg insertion-induced deletion does not affect gene expression over large regions outside the boundaries of the rearrangement.

**Table 1:** Brain microarray data for mouse chromosome 7B2-C genes^a ^in TgPWS *vs*. WT mice.

**GenBank**	**Affy ID**	**Gene**	**mRNA level^b^**		**Chr. 7 location (Mb and/or cM)**
			**TgPWS**	**WT**	
**I. Genes centromeric of the TgPWS/TgAS deletion**					
NM_008087	94337_at	*Gas2*	4.3 (3/5)	5.0 (2/5)	39.3 Mb; 26.8 cM
	94338_g_at	*Gas2*	43.1 (5/5)	42.92 (5/5)	as above
AK049836	97539_at	*Hrmt1l3*	50.2 (5/5)	44.5 (4/5)	37.25 Mb
NM_016865	103671_at	*Htatip2*	23.8 (0/5)	21.0 (0/5)	37.2 Mb
AY957576	103202_f_at	*E2F8*	35.3 (4/5)	30.4 (4/5)	36.3 Mb
	103204_r_at	*E2F8*	27.4 (1/5)	21.6 (2/5)	as above
NM_013808	103084_at	*Csrp3*	4.6 (0/5)	3.2 (0/5)	36.3 Mb
NM_013643	100406_at	*Ptpn5*	399.8 (5/5)	406.1 (5/5)	34.5 Mb
NM_016855	95304_at	*Attp*	8.5 (0/5)	8.7 (0/5)	34.3 Mb
NM_021884	94809_at	*Tsg101*	56.5 (5/5)	56.6 (5/5)	34.3 Mb
NM_013580	93103_at	*Ldh3*	1.7 (0/5)	1.3 (0/5)	34.3 Mb; 23.5 cM
**II. Genes telomeric of the TgPWS/TgAS deletion**					
NM_007390	101131_at	*Chrna7*	86.8 (0/5)	87.1 (0/5)	50.1 Mb; 30.0 cM
NM_021366	160617_at	*Klf13*	145.2 (5/5)	155.6 (5/5)	50.9 Mb
NM_018752	102251_at	*Trpm1*	77.7 (1/5)	56.7 (0/5)	51.2 Mb; 27.0 cM
BC055074	93235_at	*BB128963*	51.3 (5/5)	46.0 (5/5)	51.3 Mb
NM_026483	104022_at	*Mphosph10*	35.9 (0/5)	34.0 (0/5)	51.4 Mb
NM_028626	102022_at	*Mcee*	79.6 (5/5)	81.1 (5/5)	51.4 Mb
NM_007461	92727_at	*Apba2*	433.9 (5/5)	455.8 (5/5)	51.5 Mb; 25.5 cM
NM_009386	99935_at	*Tjp1*	185.3 (5/5)	142.5 (5/5)	52.3 Mb; 28.5 cM
D50060	101196_at	*Pace4*	78.3 (0/5)	71.2 (0/5)	52.9 Mb; 28.5 cM
NM_021336	101506_at	*Snrpa1*	131.2 (5/5)	126 (5/5)	53.1 Mb
NM_024439	94245_at	*H47*	141.8 (5/5)	144.2 (5/5)	53.1 Mb; 28.5 cM
NM_053080	98372_at	*Aldh1a3*	13.3 (0/5)	9.8 (0/5)	53.4 Mb
NM_194070	93852_at	*Mef2a*	399.9 (5/5)	417.4 (5/5)	54.3 Mb; 33.0 cM
AF056187	102224_at	*Igf1r*	381.6 (5/5)	339.6 (5/5)	55.0 Mb; 33.0 cM

^a ^The primary microarray data from this experiment were reported in reference 15. As summarized in Fig. 1A, reference 15 included the data for the imprinted genes within the TgPWS/TgAS deletion that are on the MG-U74Av2 chip (*Snrpn*, *Ndn*, *Magel2*, *Mkrn3 *and *Ube3a*) and for non-imprinted genes within the deletion (*Cyfip1*, *Herc2*, and *Gabrb3*, although the latter transcript was not detectable; the *p *gene is also represented on the chip although not detectable in TgPWS or WT mice, and not previously reported). Additionally, a cluster of chromosome 18B3 genes were found to be abnormally expressed in TgPWS mice [15]. ^b ^mRNA levels are presented as the medians of Signal (relative abundance of a transcript), for TgPWS (n = 5) and WT (n = 5), with the number of calls as "detected" out of 5 comparison experiments in parentheses.

**Figure 3:**
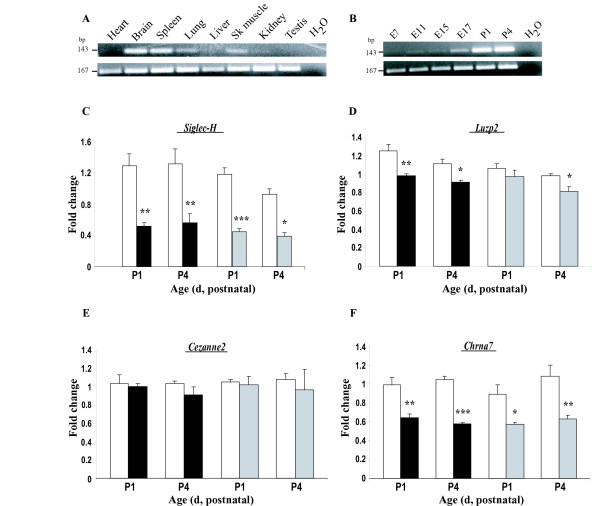
**Gene expression analyses for loci at or near the TgPWS/TgAS deletion breakpoints**. **(A) Tissue expression of *Siglec-H***. RT-PCR using a cDNA mouse panel shows that *Siglec-H *(143-bp band) is strongly expressed in adult brain and spleen, with moderate expression in lung and skeletal muscle tissues, but is low or absent in heart, liver, kidney and testis. *RNApolII *(167-bp) gene expression was used as a control for both (A) and (B). **(B) Developmental *Siglec-H *expression**. Very low or absent *Siglec-H *expression is detected by RT-PCR at E7-E15 with weak expression at late gestational stages (E17). After birth, *SiglecH *expression is robustly detected in brain at P1 and P4. **(C-F) Relative quantification of brain mRNA levels for (C) *Siglec-H*, (D) *Luzp2*, (E) *Cezanne2 *and (F) *Chrna7 ***was performed by QRT-PCR in two groups of 5 TgPWS (black bars) or 5 TgAS (gray bars) and 5 WT (white bars) mice at P1 and in 4 TgPWS or 4 TgAS and 4 WT mice at P4. All values are presented as the means ± SE: * *P *≤ 0.05; ** *P *≤ 0.001; *** *P *≤ 0.0001, significant differences between WT and TgPWS or TgAS at the indicated time points (Independent samples t-test). **(C) *Siglec-H ***mRNA levels are decreased by ~2-fold in TgPWS and TgAS compared with WT at P1 and P4. *SiglecH *mRNA levels in TgPWS *vs*. WT were 0.52 ± 0.04 *vs*. 1.29 ± 0.15 (*P *= 0.002) and 0.56 ± 0.12 vs. 1.31 ± 0.19 (*P *= 0.01) at P1 and P4, respectively. *Siglec-H *mRNA levels were 0.45 ± 0.03 in TgAS compared with 1.18 ± 0.08 in WT at P1 (*P *= 0.0002) and 0.39 ± 0.04 compared with 0.92 ± 0.07 at P4 (*P *= 0.0008). **(D) *Luzp2 ***mRNA levels in TgPWS *vs*. WT were 0.98 ± 0.02 *vs*. 1.25 ± 0.07 (*P *= 0.009) and 0.91 ± 0.02 *vs*. 1.11 ± 0.05 (*P *= 0.01) at P1 and P4, respectively. In TgAS *vs*. WT mice, mRNA levels were 0.97 ± 0.07 *vs*. 1.06 ± 0.05 (*P *= 0.3) at P1 and 0.81 ± 0.05 *vs*. 0.98 ± 0.02 (*P *= 0.01) at P4. **(E) *Cezanne2 ***shows no difference in expression in TgPWS or TgAS and WT mice. At P1, mRNA levels were 1.01 ± 0.03 for TgPWS *vs*. 1.04 ± 0.1 for WT (*P *= 0.8) and 1.03 ± 0.09 for TgAS *vs*. 1.06 ± 0.03 for WT (*P *= 0.7). At P4, C*ezanne2 *mRNA levels were 0.92 ± 0.08 for TgPWS *vs*. 1.04 ± 0.03 for WT (*P *= 0.2) and 0.97 ± 0.23 for TgAS *vs*. 1.09 ± 0.06 for WT (*P *= 0.6). **(F) *Chrna7 ***mRNA levels are ~0.6-fold in TgPWS and TgAS compared with WT mouse brain. At P1 the mRNA levels were 0.64 ± 0.04 in TgPWS *vs*. 0.99 ± 0.08 in WT (*P *= 0.005) and 0.57 ± 0.02 in TgAS *vs*. 0.89 ± 0.1 in WT (*P *= 0.01), while at P4 mRNA levels for *Chrna7 *were 0.58 ± 0.01 for TgPWS *vs*. 1.05 ± 0.03 for WT (*P *< 0.0001) and 0.63 ± 0.04 for TgAS *vs*. 1.08 ± 0.12 for WT (*P *= 0.005).

We next assessed quantitative (Q) expression of genes that map in the proximity of the known deleted loci at the centromeric [14,15] and telomeric [14] ends of the deletion (Fig. 1A). At the centromeric end of the deletion, the *Siglec-H *gene maps just proximal of *Tubgcp5 *and is predicted to have five exons (Fig. 1C), with the rare feature of having the stop codon in the penultimate exon 4 [19]. Since there is no information regarding the pattern of expression for *Siglec-H *[20,21], we first assessed this using RT-PCR (Fig. 3A,B). In agreement with the general signaling function of *Siglec *genes in the haemopoietic, immune and nervous systems [22], *Siglec-H *was predominantly expressed in brain and spleen (Fig. 3A). Less robust expression was seen in lung and skeletal muscle, while there was little or no amplification in heart, liver, kidney and testis (Fig. 3A). A postnatal role for *Siglec-H *expression was suggested by the lack of amplification at early embryonic stages with increased expression after birth (Fig. 3B), as also found for ESTs in Unigene (data not shown).

By QRT-PCR, *Siglec-H *showed 2-fold decreased mRNA levels in the brain of both TgPWS and TgAS mouse models at P1 and P4 (*P *< 0.01; Fig. 3C), indicating that this gene appears to be within the TgPWS/TgAS deletion (Fig. 1A). It may also be noted that the 0.5-fold expression level in TgPWS and TgAS mice compared with WT indicates that *Siglec-H *is not an imprinted gene. The next gene centromeric of this, *Luzp2*, showed ~0.8-fold decreased mRNA levels in TgPWS brain at P1 and P4 and in TgAS brain at P4 (*P *< 0.05) (Fig. 3D). At P1 in TgAS, *Luzp2 *brain mRNA levels were decreased to 0.91-fold *vs*. WT (*P *= 0.3). Combined, the presence of the TgPWS/TgAS deletion resulted in a *Luzp2 *mRNA level in neonatal brain that is significantly (0.83-fold, *P *= 0.0002) less than in WT. We conclude that the *Luzp2 *gene is intact at the centromeric end of the TgPWS/TgAS deletion but suggest a model in which a neuronal regulatory element is deleted that accounts for ~33% of *Luzp2 *expression from the deleted allele (see Discussion for a description of this and alternative models).

At the telomeric end of the deletion, expression of *Cezanne2*, which maps just centromeric of *Klf13 *(Fig. 1D), was unchanged by QRT-PCR in TgPWS and TgAS brain compared with WT mice (Fig. 3E), and thus this gene is intact in the TgPWS/TgAS deletion mouse model. The only remaining gene between *Cezanne2 *(intact in TgPWS/TgAS mice) and *Frat3 *(deleted [13]) is *Chrna7 *(Fig. 1D), suggesting that the deletion breakpoint might lie in the vicinity of this gene. *Chrna7 *is orientated with a telomeric 5' end (Fig. 1D). Using QRT-PCR for *Chrna7 *and primers spanning exon 2 to exon 3, we found ~1.7-fold decreased *Chrna7 *mRNA levels in both TgPWS and TgAS brain at P1 and P4 (*P *< 0.05) (Fig. 3F). Since the 5' end of *Chrna7 *is not deleted in the TgPWS/TgAS mouse model (see below, which is also consistent with the normal FISH signal for BAC RP23-16A4 reported above), the finding of ~60% total mRNA levels suggests that only ~20% of the usual level of *Chrna7 *mRNA transcripts are generated from the deleted chromosome in TgPWS and TgAS mouse brain. The 80% reduction in expression *in cis *suggests that the deletion removes essential regulatory sequences (see Discussion).

### Fine-mapping of the centromeric TgPWS/TgAS deletion breakpoint

The gene expression data described above suggest that *Siglec-H *is deleted in TgPWS and TgAS mice while *Luzp2 *is intact but shows slightly yet significantly reduced expression levels. In order to further delineate the centromeric breakpoint of the deletion and a potential mechanism for the ~33% reduction in *Luzp2 *expression from the deletion allele, we examined the genome sequence of BAC RP24-426A19 that lies between *Siglec-H *and *Luzp2 *(Fig. 1C) and that was identified above by FISH as partially deleted in TgPWS/TgAS mice. There are no exons for any gene encoded in this BAC. Nevertheless, using BLAST of RepeatMasked RP24-426A19 DNA sequence against the non-redundant GenBank database, extending from the centromeric end of BAC RP24-426A19 we identified five non-coding conserved sequences (NCCS1-5) at positions ~30.1, 39.9, 68.8, 107.3, and 134.5 kb, respectively (Fig. 1C). NCCS1-5 are 250-bp, 68-bp, 188-bp, 104-bp, and 157-bp in length and show 82%, 91%, 81%, 82% and 83% identity with human, respectively. As in mouse, the five NCCS elements in human are located adjacent to *LUZP2 *and map in chromosome 11p14.3 (Fig. 1B). All five NCCS elements are likely to be involved in the regulation of *LUZP2*, while other flanking genes are unlikely to be regulated by or to relate to NCCS1-5, as the next most centromeric gene (*Gas2*) maps 3.2 Mb from *Luzp2 *while the genes more telomeric are either non-syntenic in human (Fig. 1B), mapping to human chromosome 15q, or represent a rodent-specific acquisition (*Siglec-H*; see Discussion).

**Figure 4:**
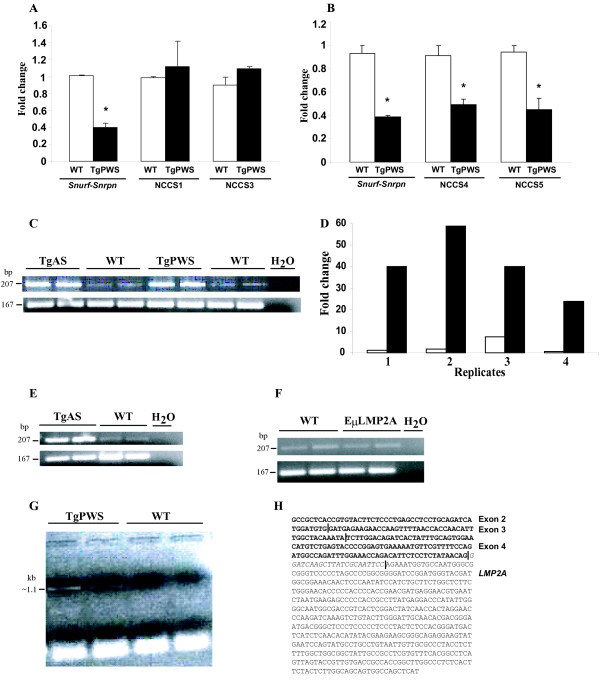
**Mapping of centromeric and telomeric TgPWS/TgAS deletion breakpoints and tissue specific up-regulation of *Chrna7***. **(A) Q-PCR for the *Snurf-Snrpn *promoter, NCCS1 and NCCS3 relative to *Gapdh *intron 1**. The amount of *Snurf-Snrpn *DNA was 1.01 ± 0.01 for the WT compared with 0.40 ± 0.05 for the TgPWS samples, *P *= 0.006. For NCCS1, DNA fold was 0.98 ± 0.02 in WT *vs*. 1.12 ± 0.30 in TgPWS, *P *= 0.68. For NCCS3, DNA fold was 0.90 ± 0.09 in WT compared with 1.09 ± 0.03 in TgPWS mice, *P *= 0.20. **(B) Q-PCR for NCCS4 and NCCS5 relative to *Gapdh *intron 1**. *Snurf-Snrpn *DNA-fold was 0.40 ± 0.07 in TgPWS *vs*. 0.90 ± 0.1 in WT mice, *P *= 0.02. The amount of DNA for NCCS4 was 0.50 ± 0.05 in TgPWS and 0.90 ± 0.1 in WT mice, *P *= 0.05. For NCCS5, DNA-fold was 0.45 ± 0.1 in TgPWS and 0.94 ± 0.06 in WT mice, *P *= 0.05. **(C) *Chrna7 *expression (207-bp) is dramatically upregulated in liver **of TgPWS and TgAS at P1 compared with WT mice by RT-PCR. *RNApolII *expression (167-bp) was used as a control in (C), (E) and (F). **(D) Relative quantification by QRT-PCR of *Chrna7 *liver mRNA levels **shows a ≥15-fold increased expression in TgPWS (black bars) *vs*. WT (open bars) mice. Individual values for *Chrna7 *mRNA expression of 4 TgPWS and 4 WT mice at P1 are shown. The average mRNA expression was 40.76 ± 14.30 for TgPWS and 2.70 ± 3.21 for WT, *P *= 0.002. **(E) Up-regulation of *Chrna7 *expression in TgAS spleen **compared with WT at P1 by RT-PCR. **(F) *Chrna7 *is normally expressed in P1 liver from E_μ_LMP2A transgenic mice **compared with WT liver. **(G) A *Chrna7*-*LMP2A *fusion transcript is identified by RT-PCR in TgPWS brain **(lanes 1,2) but not in WT (lanes 3,4). **(H) DNA sequence of the *Chrna7*-*LMP2A *fusion cDNA**. Vertical lines mark the limit between exons 2, 3 and 4 of *Chrna7 *(bold font) and the 5' end of *LMP2A *sequence, while the 22-nt in italics is a transgene-specific sequence at the 5' end of the *LMP2A *exon.

PCR primers were designed from four of the NCCS elements and Q-PCR of DNA from TgPWS and WT mice was used to examine the relative amount of each NCCS with respect to intron 1 of *Gapdh*, which is intact in all mice studied. As a deletion control, we similarly examined by Q-PCR the *Snurf-Snrpn *promoter, which as expected showed a 0.4 to 0.44-fold relative level in TgPWS *vs*. WT mice (Fig. 4A,4B). Whereas there was no difference between TgPWS and WT mice for NCCS1 and NCCS3 (Fig. 4A), indicating that NCCS1, NCCS2 and NCCS3 are intact in TgPWS mice, Q-PCR for NCCS4 and NCCS5 identified 1.8-fold and 2.1-fold lower levels for TgPWS *vs*. WT, respectively (Fig. 4B), indicating that these are deleted from one allele in TgPWS mice. Therefore, the centromeric breakpoint of the TgPWS/TgAS ~5 Mb deletion lies in the 38.5 kb region between NCCS3 and NCCS4.

### Up-regulation of Chrna7 allows mapping the telomeric TgPWS/TgAS deletion breakpoint

In contrast to the results shown above for *Chrna7 *expression in brain, regular RT-PCR for *Chrna7 *in newborn liver showed a dramatic increased expression in TgPWS and TgAS compared with WT (Fig. 4C). Indeed, quantification of liver *Chrna7 *expression by QRT-PCR revealed ~15-fold increased mRNA levels in TgPWS mice (*P *= 0.002) (Fig. 4D). A similar up-regulation of *Chrna7 *expression was found by RT-PCR in spleen tissues of TgAS mice (Fig. 4E). However, E_μ_LMP2A control mice with B-cell lineage expression of *LMP2A *showed normal expression of *Chrna7 *in newborn liver (Fig. 4F). Combined, these data on two different *LMP2A *transgene models indicates that *LMP2A *expression in B cells does not upregulate *Chrna7 in trans*, and suggests that the increased expression of *Chrna7 *observed in liver and spleen from TgPWS and TgAS mice results from a *cis *effect of the transgene insertion. Since B-cell transcription of the *LMP2A *transgene in these models is driven from an immunoglobulin heavy chain gene promoter and enhancer [7,16], we hypothesized that the latter enhancer was also driving transcription of the heterologous, endogenous *Chrna7 *promoter.

**Figure 5:**
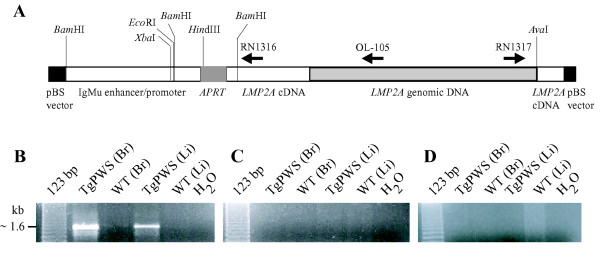
**Orientation of the *LMP2A *transgene array**. **(A) *LMP2A *transgene structure**. Restriction enzyme sites and primer location and orientation are shown. **(B) *LMP2A *transgene amplification **in TgPWS brain (Br) and liver (Li) (lanes 2,4) using primers RN1316 and RN1317 compared with the WT controls (lanes 3,5). **(C-D) **The use of **(C) **RN1316 or **(D) **RN1317 alone fails to generate PCR products in TgPWS and WT brain and liver.

To test this hypothesis, we used an exon 2 forward primer for *Chrna7 *and a reverse primer (OL-105) from inside the Tg (Fig. 5A) to perform RT-PCR on cDNA from TgPWS *vs*. WT brain, which detected a 1.1 kb product specifically in TgPWS samples (Fig. 4G). The 1.1 kb band was cloned and sequenced, identifying it as a fusion transcript encoding *Chrna7 *exons 2–4 fused to the sense strand of the *LMP2A *transgene (Fig. 4H). The latter result indicates that at least the telomeric copy of the Tg array is orientated in a 5'-3' telomere to centromere fashion. Furthermore, we can conclude that the 5' end of the *Chrna7 *locus is intact and correctly splices through exon 4 but then splices into the *LMP2A *transgene. These data strongly suggest that the telomeric breakpoint of the TgPWS/TgAS ~5 Mb deletion is in the 38.1 kb *Chrna7 *intron 4.

### Orientation of the LMP2A transgene array

To fully understand how the transgene array might affect flanking gene expression, it is necessary to define the integrity of the array and the orientation of each transgene copy and to flanking genes. To characterize the transgene array, we amplified genomic DNA from TgPWS mice and WT controls using primers from within the transgene (Fig. 5A). Amplification with either single primer (RN1316 or RN1317) did not lead to any product (Fig. 5C,D), indicating that there are no head-to-head or tail-to-tail copies of adjacent transgene inserts. In contrast, PCR using primers from adjacent copies of the transgene results in amplification of the *LMP2A *array in TgPWS DNA (Fig. 5B). These data indicate that the ~80 copies of the transgene [7] are all in the same tail-to-head orientation. Combined with the data described above on the sense strand fusion of *Chrna7 *and *LMP2A *cDNA sequences, we conclude that the entire Tg array is orientated in a 5'-3' manner from the telomeric to centromeric end.

## Discussion

### A mouse model of PWS/AS class I deletions

The TgPWS and TgAS mouse models were created by insertion of an *LMP2A *transgene array of ~80 copies [7] that fortuitously generated a deletion equivalent to those that occur in PWS and AS in the human. Although the mouse deletion was previously estimated as being ~5 Mb in length, genome sequence analysis indicates that the deleted segment could be as large as 6.8 Mb (*Siglec-H *to *Chrna7 *distance). However, there are a few gaps in the sequence, particularly in several duplicated genomic regions throughout the imprinted domain, and so the exact size of this interval remains unknown. Nevertheless, our previous [7,13-15] and present studies allow the conclusion that 13 imprinted and 11 non-imprinted genes are included within the mouse PWS/AS-deletion region (see Fig. 1A). In addition to spanning all the PWS- and AS-homologous paternally and maternally expressed genes, respectively, the TgPWS/TgAS deletion in mice also includes the homologs of typically deleted non-imprinted genes (ie., *Gabrb3*, *Gabra5*, *Gabrg3*, *p*/*Oca2*, and *Herc2*) as well as those of the PWS/AS genes (*Nipa1*, *Nipa2*, *Cyfip1 *and *Tubgcp5*) that define the deletion as equivalent to human class I deletions [1,4]. Although the classic PWS or AS clinical phenotype is similar in both class I and class II deletions, respectively, a more severe neurobehavioral phenotype has been described for class I compared with class II deletion patients as well as for deletion *vs*. UPD patients in each syndrome [23,24]. The TgPWS and TgAS deletion mouse models may therefore prove useful to compare the neurobehavioral phenotype to the UPD or ID PWS [5,6] or *Ube3a *gene mutation AS [11,12] mouse models.

Although the TgPWS/TgAS mouse models show reduced expression of several other genes, including *Siglec-H*, *Luzp2 *and *Chrna7*, as compared to the human PWS and AS class I deletions, none of these additional genes are likely to contribute to the TgPWS and TgAS phenotypes. For example, since *Siglec-H *is a recent evolutionary addition in the rodent genome [20], its 50% reduction in expression levels due to hemizygosity at the centromeric end of the deletion is unlikely to have a phenotypic effect. Likewise, the ~17% reduction in overall expression of *Luzp2 *observed in brain of TgPWS/TgAS mice is unlikely to be functionally important, since complete loss of *Luzp2 *does not lead to any specific phenotype [25]. Although *Chrna7 *is another gene partially deleted in TgPWS/TgAS mice and the human ortholog maps in 15q13, the latter is outside the PWS/AS deletions and the TgPWS/TgAS phenotype is unlikely to be affected by *Chrna7 *hemizygosity, since *Chrna7 *heterozygous knockout mice show no abnormal phenotypes and even *Chrna7*-/- null mice have only mild skin and reproductive phenotypes [26-29].

### Implications for evolutionary acquisition of new genes and breakpoint mechanisms

Our study also characterized expression of a recently identified gene, *Siglec-H*, which belongs to the Siglec gene family encoding immunoglobulin-like lectins that act as extracellular receptors for sialic acid residues of glycan chains [20,21]. The majority of *Siglec *genes map in a single *CD33 *(*Siglec-3*)-related cluster in human 19q13 and the syntenic mouse chromosome 7B2 region [20,21]. In contrast, we show here that *Siglec-H*, which is rodent-specific and has no primate ortholog [21], lies adjacent to the mouse PWS/AS-homologous region at a position 12.3 Mb from its ancestral location. It seems likely that Siglec-H has similar postnatal immunological functions as other Siglecs, given its robust expression in spleen. However, in contrast with the restricted expression of most other *Siglec *genes to the haematopoietic and immune systems, *Siglec-H *is also transcribed at high levels in postnatal brain. Although conjectural, it is tempting to speculate that neuronal expression of *Siglec-H *may have arisen due to an evolutionary positioning adjacent to single (*Luzp2*) [25] or clustered (ie., *Tubgcp5*, *Cyfip1*, *Nipa2*, *Nipa1*) [14] genes expressed at high levels in the nervous system, such that *Siglec-H *transcription may be under control of a neuronal enhancer for one or more of these genes.

Intriguingly, both the centromeric and telomeric TgPWS/TgAS deletion breakpoints map at or close to the positions of chromosomal evolutionary breakpoints, and at each of the latter two positions there are also rodent-specific gene duplication-insertions. At the centromeric end, the TgPWS/TgAS deletion breakpoint lies immediately adjacent to *Siglec-H*, which as discussed above arose in rodents by a genomic duplication from a precursor gene located ~12 Mb away. The evolutionary insertion of *Siglec-H *between *Tubgcp5 *and *Luzp2 *occurs right at the boundary of synteny with human chromosome 15q11.2-q13 and 11p14.3-p15.1, respectively (Fig. 1B). Moreover, the *TUBGCP5 *ortholog also lies within a few kilobases of a primate evolutionary breakpoint that translocated the *TUBGCP5 *– *CYFIP1*-*NIPA2*-*NIPA1*-*HERC2*-duplicon cluster to 15q11.2 from an ancestral 15q13 location [14]. Although the telomeric TgPWS/TgAS deletion breakpoint within *Chrna7 *is not right at an evolutionary breakpoint, this gene also lies adjacent to an evolutionary breakpoint separating genes whose human orthologs map to the PWS imprinted domain in 15q11.2 and a point 8.7 Mb away in 15q13.3 (Fig. 1B). At this position also, there are species-specific duplications in mouse and human, with the retrotransposed *Frat3 *limited to rodents [13] or the *HERC2*- and flanking duplicons in primates [5,14]. Other authors have also noted the apparent congruity of chromosome rearrangement and evolutionary breakpoints [14,30-32], suggesting that currently unknown chromosomal structural features in these regions may play recombinogenic roles.

### Effect of the transgene or deletion on gene expression: mapping of putative neuronal enhancers

**Figure 6:**
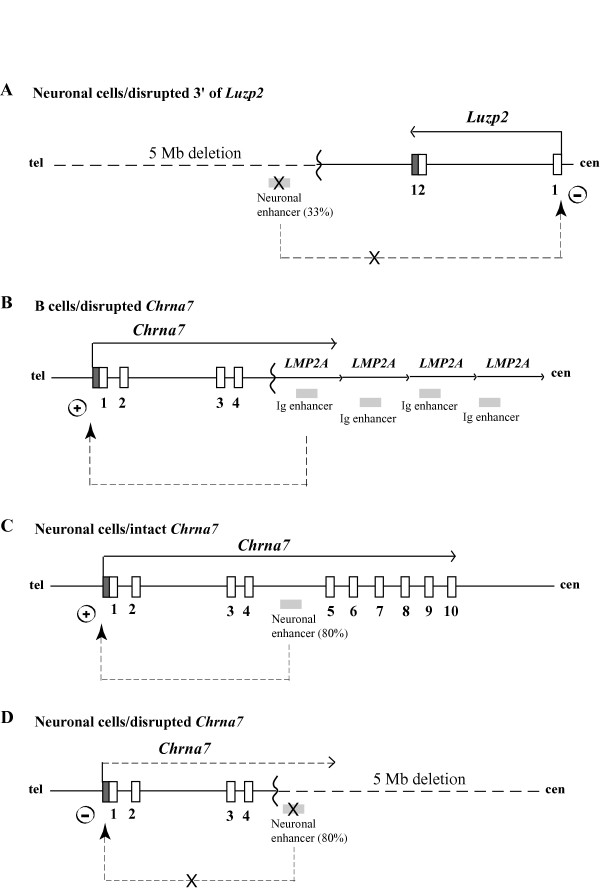
**Enhancer models for tissue-specific expression of *Luzp2 *and *Chrna7***. **(A) Deletion of a putative neuronal enhancer 3' of *Luzp2 *lowers expression levels of the gene**. Symbols are: white boxes, exons; grey vertical box, 3'-untranslated region (UTR); arrow, transcriptional orientation; zig-zag line, transgene insertion-deletion breakpoint; grey horizontal box, enhancer; dashed arrow, neuronal enhancer contributing ~33% to the *Luzp2 *allelic promoter activity; X, block to enhancer function. Only the first and last exons of *Luzp2 *are shown. **(B) Activation in TgPWS and TgAS mice of the *Chrna7 *promoter *in cis *by the immunoglobulin (Ig) enhancer from the *LMP2A *transgene active in B lymphocytes**. Symbols are as for Fig. 6A, except: grey vertical box, 5'-untranslated region (UTR); dashed arrow, Ig enhancer upregulation of *Chrna7 *transcription. **(C) Activation of the *Chrna7 *promoter in WT mice by a putative neuronal enhancer**. The location of the enhancer is drawn arbitrarily but must map 3' to the TgPWS/TgAS deletion breakpoint in intron 4. Symbols are as for Fig. 6B, except: dashed arrow, neuronal enhancer for upregulation (~80%) of normal *Chrna7 *transcription. **(D) Deletion of a neuronal enhancer in TgPWS and TgAS mice virtually silences *Chrna7 *expression *in cis***. Symbols are as for Fig. 6A and 6C.

Transgene insertions may be accompanied by other chromosomal rearrangements such as deletions (this paper; [33]), duplications [34], inversions [35], translocations [36], or combinations of rearrangements [36,37]. These events can induce alterations of gene expression over large chromosomal regions [33], or the transgene itself can do so [38]. Extensive analysis of genes flanking the TgPWS/TgAS transgene insertion demonstrated no other rearrangements other than the PWS/AS-region deletion and that gene expression over large regions outside the immediate flanking genes was not affected. In contrast, expression from the first gene promoter outside either end of the transgene insertion-deletion was affected. For example, the 5' promoter and 3' end of *Luzp2 *are ~600 kb and 110–140 kb centromeric to the *LMP2A *transgene array-deletion breakpoint, respectively, and this gene shows a significantly reduced expression in TgPWS and TgAS mice. Although at present we cannot fully exclude three models that might lower the *Luzp2 *transcriptional level *in cis *in brain, which invoke either (i) a non-specific disruption of chromatin, (ii) a weak silencing effect from the transgene tandem array [45], or (iii) extended antisense transcripts from the transgene array given that the 5'-3' orientation of the *LMP2A *array is opposite to the *Luzp2 *direction of transcription, we feel that these are less likely than a fourth model that has additional experimental support. We propose that the 33% reduction in expression of *Luzp2 *from the deletion allele is most likely explained by deletion of a neuronal enhancer (Fig. 6A). Consistent with this model, we identified two NCCS elements (NCCS4 and NCCS5) conserved in all eutherian mammals sequenced to date and deleted in TgPWS and TgAS mice, that represent strong candidates to be the neuronal enhancer. As noted above, these elements map to the homologous chromosome location in human but are almost 4 Mb from the next most centromeric gene (*Gas2*) while other flanking genes in mouse are non-syntenic (*Tubgcp5*) or not present (*Siglec-H*) in human (Fig. 1B). Numerous NCCSs occur within mammalian genomes [39,40], and while the majority of these have no known function, many do act as enhancer elements [41-43]. Future *in vitro *and *in vivo *studies [44] will be able to examine this enhancer model for *Luzp2*.

At the telomeric end of the deletion, expression of *Chrna7 *was dramatically upregulated in liver and spleen, but not brain, due to a tissue-specific positional effect of the transgene. We propose that the transgene immunogobulin Mu enhancer acts in B cells to promote transcription *in cis *from the heterologous *Chrna7 *promoter (Fig. 6B). Similarly, transgene regulatory elements 1 Mb away have been shown to activate *Sox9 *[38]. Moreover, our data combined with cloning of a 5'-*Chrna7*-*LMP2A *fusion transcript clearly demonstrates that the *Chrna7 *promoter is intact and fully capable of high levels of enhancer driven activity on the deletion allele in TgPWS/TgAS mice.

Perhaps the most intriguing finding of this study was our serendipitous discovery that a putative neuronal enhancer for the *Chrna7 *gene maps within the telomeric end of the TgPWS and TgAS deletion. In TgPWS and TgAS mice, the *Chrna7 *mRNA level in brain from the deletion chromosome 7 falls to 20% of normal levels despite the fact that the 5' promoter is not deleted. Non-enhancer models appear unlikely to explain this data: for example, models invoking (i) non-specific disruption of chromatin or (ii) a silencing effect from the transgene tandem array [45] are unlikely to account for the specific ~80% reduction in *cis *expression levels in TgPWS and TgAS mouse brain, while allowing a dramatic activation of the same promoter in immune tissues, and, finally (iii) *Chrna7 *is transcribed from upstream and in the same orientation as the transgene array, so that an antisense mechanism is not valid. Therefore, we propose that sequences that map 3' of the deletion breakpoint in *Chrna7 *intron 4 are critical for *Chrna7 *expression in neurons (Fig. 6C), and it is the deletion of this putative neuronal enhancer that accounts for the reduced *Chrna7 *expression in brain of TgPWS and TgAS mice (Fig. 6D). Similar analyses of *Chrna7 *gene expression in other tissues of TgPWS and TgAS mice, such as the neuromuscular junction, heart, and skin, will allow further assessment of the tissue specificity of the proposed enhancer. Interestingly, our data in brain and other tissues also imply that the *Chrna7 *promoter *in vivo *only has basal transcriptional activity whereas for high levels of transcription, *Chrna7 *requires activation by a tissue-specific enhancer(s). In support of this model, we recently identified as candidate enhancer elements two NCCSs within *Chrna7 *intron 4, whereas no other conserved non-coding sequences other than the minimal promoter occur in a 412 kb domain starting 18 kb 5' of *Chrna7 *and extending 3' of the gene towards the mouse PWS-region imprinted domain (R.D.N., K.C., and M.S., unpublished data). We are currently mapping the *Chrna7 *NCCS elements against the TgPWS/TgAS deletion endpoints, while *in vitro *and *in vivo *approaches will be needed for analysis of function as candidate neuronal transcriptional enhancers and/or for other cell types.

## Conclusion

In this study, we used a variety of molecular cytological and genetic technologies to map the transgene insertion-chromosome deletion breakpoints of a mouse model of PWS and AS to ~38 kb regions between the *Luzp2 *and *Siglec-H *genes at the centromeric end and within *Chrna7 *intron 4 at the telomeric end, respectively. Gene expression analyses then allowed us to demonstrate that genes extending out a further 9.1- or 5.6-Mb centromeric or telomeric of the deletion, respectively, are not affected by either the deletion or insertion of the transgene tandem array. In contrast, transcription of genes at (*Chrna7*) or flanking (*Luzp2*) the transgene insertion-deletion breakpoints are affected by the disruption of normal chromosome architecture, with positional effects leading to up- or down-regulation dependent on the tissue-specificity and locations of enhancers within the transgene or putatively removed by the TgPWS/TgAS chromosome deletion. Using *Luzp2 *as an example, we demonstrate that analysis of phylogenetically conserved sequences and consideration of synteny allows the fine mapping of a putative neuronal enhancer element(s), while a similar model likely also applies for *Chrna7*. In human, *CHRNA7 *is of significant interest as a schizophrenia candidate gene with both genetic and functional support [46-48]. Nevertheless, the molecular basis of its candidacy remains uncertain since rare coding and *in vitro *characterized promoter variants in *CHRNA7 *[49,50] may be insufficient to account for deficient function *in vivo *[51], particularly in light of our observations that expression of *Chrna7 in vivo *requires a major participation of enhancer function. Identification of a neuronal enhancer and further studies in the human to examine for genetic variation and potential mutations might provide an explanation for a role of *CHRNA7 *as a schizophrenia-susceptibility gene.

## Methods

### Animals

We used three mouse models all of which have an *LMP2A *transgene insertion. In the TgPWS and TgAS models, the insertion of an *LMP2A *transgene array replaced the PWS/AS-homologous region in chromosome 7B/C [7,9,15] while the E_μ_LMP2A mice have B-cell lineage expression of *LMP2A *[16]. WT littermates were used as controls in each experiment. The University of Pennsylvania Institutional Animal Care and Use Committee approved all animal experiments. All animals were bred and genotyped as described [7,16].

### BAC contig assembly and fluorescence in situ hybridization (FISH) studies

Splenocytes from TgAS mice were cultured for 48 hr in the presence of concanavalin A for stimulation of cell proliferation. After harvesting, cells were treated with hypotonic buffer (0.075 M KCl) for 15 min, chromosomes were fixed with methanol-acetic acid (3:1) and spread onto slides [17]. For some probes, fibroblast cell lines from TgPWS mice were used. BAC DNA was labeled with biotin or digoxigenin and hybridized as previously described [18]. Image acquisition was performed using fluorescence microscopy, acquiring grayscale images using separate filters for each fluorophore followed by digital merging and pseudocoloring. BAC RP22-434N7, spanning the mouse chromosome 7 tyrosinase gene, was used as a control probe to identify both chromosome 7 homologues.

The chromosome 7B/C BAC contigs were generated by standard bioinformatics analyses using Ensembl [52] and BLAST [53]. The position and overlap of each BAC clone was determined by BLAST using gene cDNA/EST sequences, BAC end sequences, and STS sequences. Previously described BACs are RP23-266F22 [13], RP23-256L9, RP23-195C6, RP24-396N13 and RP24-354P8 [14]. It may be noted that we previously incorrectly assumed the position of *Trpm1 *(formerly *Mlsn1*) [7], whereas analysis of genomic sequence data now clearly place this locus telomeric to the TgPWS/TgAS deletion (see Fig. 1A).

### Gene expression analyses

#### Microarray analysis

Brain global gene expression was assessed in five TgPWS and five WT male CD-1 sibs at P1 using the Affymetrix Murine Genome (MG) U74Av2 Array. The microarray experiments and data processing, with identification of genes significantly altered in TgPWS brain, were performed previously (see also Table 1) [15]. In the present study, we reanalyzed the microarray data for genes identified from bioinformatics analyses (see above) as mapping adjacent to the TgPWS/TgAS deletion.

#### QRT-PCR

Brain tissues from four or five each of TgPWS, TgAS and littermate WT mice at P1 and P4 were used for most QRT-PCR experiments. Liver *Chrna7 *expression was determined in four TgPWS and four WT mice at P1. QRT-PCR analysis was performed as described [15]. Briefly, total RNA was extracted using TRIzol (Invitrogen) and reverse-transcribed using Superscript First-Strand Synthesis System (Invitrogen). For all reactions, each sample was loaded in triplicate and SYBR green was used as the fluorescent dye. *Gapdh *was used as an internal control. Relative quantification of gene expression was carried out using a PRISM 7000 Sequence Detection System (Applied Biosystems Inc.) and data was processed using a comparative C_T _method [15]. A t-test for independent samples (Analyze-it) was used to generate two-tailed *P *statistics for each experiment. PCR primers were: *Cezanne2 *(exons 11–12): RN2262: 5'-TGATTCACAAGCTCCCCTAGCT-3' (F), RN2263: 5'-GGAGTGGACCTGGGTTCATCT-3' (R); *Chrna7 *(exons 2–3): RN2357: 5'-GCCGCTCACCGTGTACTTCT-3' (F), RN2358: 5'-GGTGGTTAAAACTTGGTTCTTCTCA-3' (R); *Luzp2 *(exons 7–8): RN2264: 5'-AAATCCAAGCCCAGCTGAAA-3' (F), RN2265: 5'-TGTTGGGCCTTAAATAACAAATCTT-3' (R); and *Siglec-H *(exons 1–2): RN2464: 5'-AGGATCTCTGTGCATGTGACAGA-3' (F), RN2465: 5'-AGGACGACCAAGCTCCAGTGT-3' (R).

#### Regular RT-PCR

Liver expression of *Chrna7 *was assessed in 2 TgPWS, 2 TgAS and 2 E_μ_LMP2A mice and WT littermates at P1. In addition, *Chrna7 *expression in spleen was determined in 2 TgAS *vs*. 2 WT mice at P1. Extraction and reverse transcription of total RNA was performed as for QRT-PCR experiments. *Chrna7 *primers (exons 1–3) were: RN2368: 5'-GGAGGCATCTGGCTGGCTCTG-3' (F), and RN2358 (R; see above). *Siglec-H *expression was performed using a Mouse Multiple Tissue cDNA Panel I (BD Bioscience Clontech) as well as WT brain at P1 and P4. *Siglec-H *primers (exons 1–2) were RN2511: 5'-GTGACAACGGTTCTTACT-3' (F) and RN2465 (R). For each gene analysis, *RNApolII *expression was used as a control: RN2318: 5'-ACTCCTTCACTCACTGTCTTCCTGTT-3' (F), RN2319: 5'-TCCTGATCTTCTGCCACCACTGT-3'(R). PCR conditions for *Chrna7*, *Siglec-H*, and *RNApolII *were initial denaturation at 94°C, 10 min, followed by 32 cycles of denaturation at 94°C for 30 sec, annealing at 55°C, 30 sec, and extension at 72°C, 30 sec, with a final extension at 72°C, 10 min, using Gold Taq DNA polymerase (ABI).

### NCCS analysis at the centromeric TgPWS/TgAS deletion breakpoint

We identified non-coding conserved sequences (NCCS) by taking BAC genomic sequence, masking repetitive sequences with RepeatMasker [54], then using BLAST analysis of the non-redundant (NR) and whole genome shotgun (wgs) databases. From BAC RP24-426A19, we identified five NCCS elements (see Results), and PCR primers for NCCS1, NCCS3, NCCS4 and NCCS5 were designed and used to quantify the DNA amount in WT and TgPWS mice by Q-PCR. Brain and liver DNA were extracted using a phenol-chloroform method. 500 ng of each DNA sample was used for Q-PCR and SYBR green was used as the fluorescent dye, with each Q-PCR reaction performed in triplicate. As an internal control for comparison of test DNA sequence, we used PCR for *Gapdh *intron 1: RN2518: 5'-GGCCGCCGCCATGT-3' (F) and RN2519: 5'-GGAAGGCCTAAGCAAGATTTCA-3' (R). In addition, primers from the *Snurf-Snrpn *promoter were used as a positive control for the deletion status in TgPWS DNA: RN2142: 5'-GCAAAAATGTGCGCATGTG-3' (F) and RN2143: 5'-CTCTCCTCTCTGCGCTAGTCTTG-3' (R). The C_T _method was used to process the data [15] and all the samples were normalized to the same WT sample. Primers for NCCS1 were: RN2515: 5'-AAATCATGAGCCAAGCCAAAA-3' (F) and RN2516: 5'-TGGCTTCCCTTATCACTTTCACA-3' (R); for NCCS3: RN2513: 5'-TTGGAACATGCAGAACAATGAAT-3' (F) and RN2514: 5'-AGGCTGCCAACCTGCAAA-3' (R); for NCCS4: RN2522: 5'-GCCACTAAATTGGATCCTTAGACATAT-3' (F) and RN2523: 5'-AAACCTGTTCCTACCCATGATAATCT-3' (R); and for NCCS5: RN2524: 5'-TCTCCCCCTAGGTCTTCTGTTTAA-3' (F) and RN2525: 5'-TGGCCAGTGATC ATGTACAGATC-3' (R).

### Fine mapping of the telomeric TgPWS/TgAS deletion breakpoint

To fine map the telomeric TgPWS/TgAS breakpoint, total RNA from TgPWS brain tissue was reverse-transcribed using the Superscript First-Strand Synthesis System (Invitrogen) with a reverse primer from inside the transgene (Fig. 5A): OL-105: 5'-CGTGTGGCTTAC CTGCTGCCAATG-3'. cDNA was amplified by PCR using the RN2357 forward primer for *Chrna7 *(exon 2) and OL-105. The amplification conditions were initial denaturation at 94°C for 10 min, followed by 37 cycles of denaturation at 94°C for 4 min, annealing at 61°C for 1 min, and extension at 72°C for 6 min, with a final extension at 72°C for 10 min, using Platinum *Pfx *DNA polymerase (Invitrogen). PCR products were cloned into the pCR2.1-TA vector using a TOPO TA cloning kit (Invitrogen) and sequenced using standard techniques (University of Pennsylvania DNA sequencing Core facility).

### Orientation of the LMP2A transgene

To determine the orientation of the *LMP2A *transgene array, three types of PCR amplifications were performed, first using forward and reverse primers located at the 3' and 5' ends of the transgene sequence, respectively (Fig. 5A): RN1316: 5'-GTTGTTTCCGCCATCGTACC-3' (R) and RN1317: 5'-TATGAGCTACTCTCTGACCC-3' (R). The other two reactions were performed using either forward or reverse primers alone. The amplification conditions were 95°C for 10 min followed by 32 cycles of denaturation at 95°C for 30 sec, annealing at 59°C for 30 sec, and extension at 72°C for 30 sec, with a final extension at 72°C for 10 min, using Platinum *Taq *Gold polymerase (ABI).

## Authors' contributions

MS prepared tissue samples, performed PCR, RT-PCR, QRT-PCR and QPCR studies, microarray and genome sequence analyses and drafted the manuscript. KCC carried out some RT-PCR experiments and performed the identification of the telomeric deletion breakpoint and DNA sequence analyses. ES performed FISH studies. JHC contributed to BAC contig assembly. TO performed transgene array analyses and designed these experiments. RL established the transgenic mouse lines and provided the E_μ_LMP2A mouse tissues. JMG designed, supervised and interpreted the FISH experiments. RDN designed the study, coordinated the project, performed genome sequence analysis, BAC contig assembly, participated in drafting and edited the manuscript.
